# Supplementary material for: FastqPuri: high-performance preprocessing of RNA-seq data
Source: BMC Bioinformatics. 2019 May 3;20:226. doi: 10.1186/s12859-019-2799-0 (PMC6500068; doi:10.1186/s12859-019-2799-0)
Supplement: Supplementary file 2 — Archive of FastqPuri. Archive containing all files needed to install and run FastqPuri v1.0.6. Date stamp March 22, 2019. (GZ 47,819 kb) [file 12859_2019_2799_MOESM2_ESM.gz › FastqPuri-1.0.6/html/stats__info_8h.html]

FastqPuri: include/stats\_info.h File Reference


FastqPuri


- include

Classes |
Typedefs |
Functions

stats\_info.h File Reference

Construct the quality report variables and update them.
More...

`#include <stdint.h>`  
`#include <stdlib.h>`  
`#include "fq_read.h"`  
`#include "defines.h"`

Include dependency graph for stats\_info.h:

This graph shows which files directly or indirectly include this file:

Go to the source code of this file.

|  |  |
| --- | --- |
| Classes | |
| struct | statsinfo |
|  | stores info needed to create the summary graphs More... |
|  | |

|  |  |
| --- | --- |
| Typedefs | |
| typedef struct statsinfo | Info |
|  | stores info needed to create the summary graphs |
|  | |

|  |  |
| --- | --- |
| Functions | |
| void | init\_info (Info \*res) |
|  | Initialization of a Info type. More... |
|  | |
| void | free\_info (Info \*res) |
|  | frees allocated memory in Info |
|  | |
| void | read\_info (Info \*res, char \*file) |
|  | Read Info from binary file. |
|  | |
| void | write\_info (Info \*res, char \*file) |
|  | Write info to binary file. |
|  | |
| void | print\_info (Info \*res, char \*infofile) |
|  | print Info to a textfile |
|  | |
| void | get\_first\_tile (Info \*res, Fq\_read \*seq) |
|  | gets first tile |
|  | |
| void | update\_info (Info \*res, Fq\_read \*seq) |
|  | updates Info with Fq\_read |
|  | |
| int | update\_ACGT\_counts (uint64\_t \*ACGT\_low, char ACGT) |
|  | update, for current tile, ACGT counts. More... |
|  | |
| void | update\_QPosTile\_table (Info \*res, Fq\_read \*seq) |
|  | update QPostile table |
|  | |
| void | update\_ACGT\_pos (uint64\_t \*ACGT\_pos, Fq\_read \*seq) |
|  | update ACGT\_pos |
|  | |
| void | resize\_info (Info \*res) |
|  | resize Info More... |
|  | |

## Detailed Description

Construct the quality report variables and update them.

Author
:   Paula Perez paula.nosp@m.pere.nosp@m.zrubi.nosp@m.o@gm.nosp@m.ail.c.nosp@m.om

Date
:   04.08.2017

## Function Documentation

## ◆ init\_info()

|  |  |  |  |  |  |
| --- | --- | --- | --- | --- | --- |
| void init\_info | ( | Info \* | *res* | ) |  |

Initialization of a Info type.

It sets: nQ, read\_len, ntiles, minQ and the dimensions of the arrays. Initializes the rest of the variables to zero and allocates memory to the arrays initializing them to 0 (calloc).

## ◆ resize\_info()

|  |  |  |  |  |  |
| --- | --- | --- | --- | --- | --- |
| void resize\_info | ( | Info \* | *res* | ) |  |

resize Info

At the end of the program, resize the structure Info, and adapt it to the actual number of tiles and the actual number of different quality values present.

## ◆ update\_ACGT\_counts()

|  |  |  |  |
| --- | --- | --- | --- |
| int update\_ACGT\_counts | ( | uint64\_t \* | *ACGT\_low*, |
|  |  | char | *ACGT* |
|  | ) |  |  |

update, for current tile, ACGT counts.

Makes update of ACGT counts for the current tile. Can be used with variables: lowQ\_ACGT\_tile and ACGT\_tile


---

Generated on Mon Mar 19 2018 23:42:01 for FastqPuri by  

 1.8.14
